# Curcumin suppresses LGR5(+) colorectal cancer stem cells by inducing autophagy and via repressing TFAP2A-mediated ECM pathway

**DOI:** 10.1007/s11418-021-01505-1

**Published:** 2021-03-13

**Authors:** Xiaohong Mao, Xin Zhang, Xiaowei Zheng, Yongwu Chen, Zixue Xuan, Ping Huang

**Affiliations:** 1 Department of Pharmacy, Zhejiang Provincial People’s Hospital, People’s Hospital of Hangzhou Medical College, Hangzhou, 310014 China; 2 Department of Pathology, Zhejiang Provincial People’s Hospital, People’s Hospital of Hangzhou Medical College, Hangzhou, 310014 China; 3 Department of Pharmacy, Zhejiang Cancer Hospital, Hangzhou, 310022 China; 4 Department of Pharmacy, Division of Life Sciences and Medicine, The First Affiliated Hospital of USTC, University of Science and Technology of China, Hefei, 230036 China

**Keywords:** Colorectal cancer, Curcumin, Cancer stem cells, Autophagy, LGR5, TFAP2A

## Abstract

**Abstract:**

Colorectal cancer stem cells (CSCs) have the potential for self-renewal, proliferation, and differentiation. And LGR5 is a stem cell marker gene of colorectal cancer. Curcumin can suppress oncogenicity of many cancer cells, yet the effect and mechanism of curcumin in LGR5(+) colorectal cancer stem cells (CSCs) have not been studied. In this study, we studied the effect of curcumin on LGR5(+) colorectal CSCs using the experiments of tumorsphere formation, cell viability and cell apoptosis. Then autophagy analysis, RNA-Seq, and real-time PCR were used to identify the mechanism responsible for the inhibition of LGR5(+) colorectal CSCs. Our results showed that curcumin inhibited tumorsphere formation, decreased cell viability in a dose-dependent manner, and also promoted apoptosis of LGR5(+) colorectal CSCs. Next, we found curcumin induced autophagy of LGR5(+) colorectal CSCs. When LGR5(+) colorectal CSCs were co-treated with curcumin and the autophagy inhibitor (hydroxychloroquine), curcumin-induced cell proliferation inhibition decreased. In addition, we also found that curcumin inhibited the extracellular matrix (ECM)-receptor interaction pathway via the downregulation of the following genes: GP1BB, COL9A3, COMP, AGRN, ITGB4, LAMA5, COL2A1, ITGB6, ITGA1, and TNC. Further, these genes were transcriptionally regulated by TFAP2A, and the high expression of TFAP2A was associated with poor prognosis in colorectal cancer. In conclusion, curcumin suppressed LGR5(+) colorectal CSCs, potentially by inducing autophagy and repressing the oncogenic TFAP2A-mediated ECM pathway.

**Graphic abstract:**

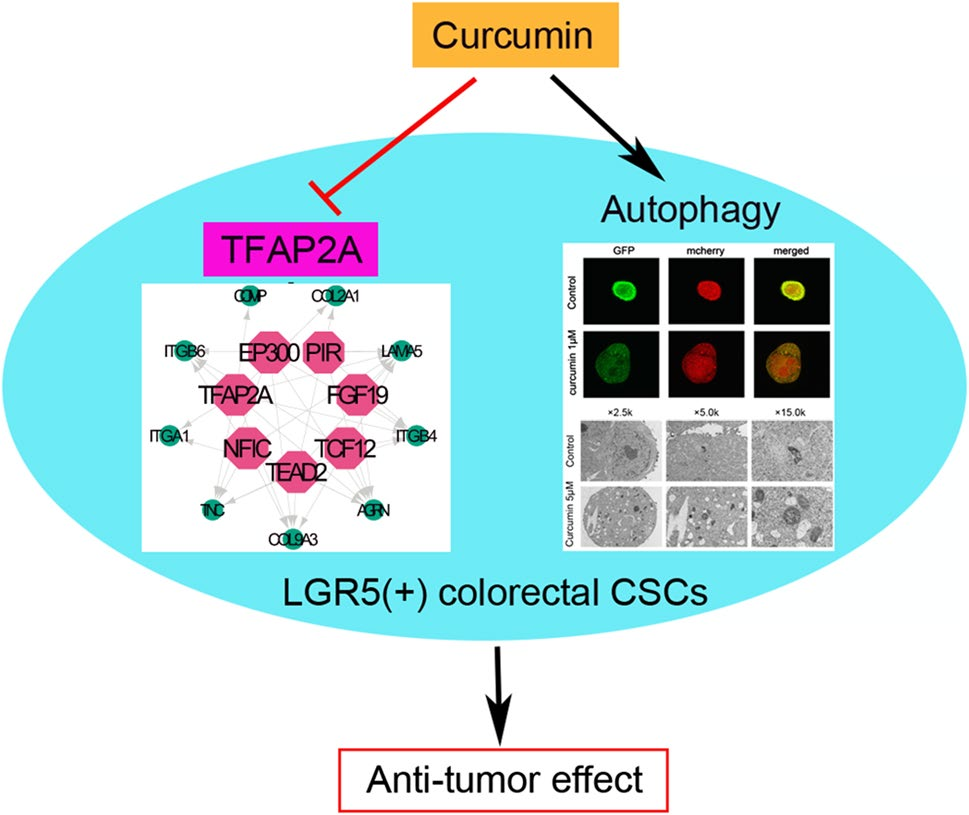

## Introduction

Colorectal cancer (CRC) is the world’s fourth most deadly cancer, and the second and third most common cancer diagnosed in women and men, respectively [1]. Advanced stage or metastatic CRC have limited sensitivity to conventional antitumor agents, because a subpopulation of colorectal cancer stem cells (CSCs) displays self-renewal, chemoresistance, and metastatic potential [2–4]. At present, more studies have confirmed that CSCs adversely affect the outcomes of CRC patients, yet agents targeting CSCs are not currently available in clinic [5].

Curcumin is a polyphenol extracting from *Curcuma longa.* It has been demonstrated that curcumin possesses extensive therapeutic activities against multiple ailments, such as inflammation, metabolic syndrome, liver disease, arthritis, and neurodegenerative disease [6]. In the recent years, researchers found that curcumin has played an important role in cancer prevention and treatment [7–9]. For instance, curcumin induced apoptosis of castration-resistant prostate cancer cells, partially dependent on its iron-chelating properties [10]; curcumin could overcome gefitinib-resistance in nonsmall-cell lung cancer cells via inducing autophagy-related cell death [11]. Other studies indicated curcumin suppressed oncogenicity of human colon cancer cells, through covalent modification of SIRT1 at the cysteine 67 residue and the proteasomal degradation of oncogenic SIRT1 [12]. However, there have been no studies elucidating the effect of curcumin and its related mechanisms in colorectal CSCs.

In addition, recent studies have revealed that LGR5 is one of the few cell surface markers used to identify and isolate actively cycling stem cells in the colonic crypt [13]. Furthermore, it is widely known that colorectal CSCs are best studied in vitro using human LGR5(+) colorectal cancer cells [14]. For example, Morgan RG thought there is the plasticity or redundancy of LGR5 + cells in intestinal cancer progression and targeting LGR5 may be a great therapeutical strategy for CRC.

To investigate the effects and the mechanism of curcumin on LGR5(+) colorectal CSCs, we used *vitro* studies including the tumorsphere formation, cell viability and cell apoptosis, to illuminate the effect of curcumin on LGR5(+) colorectal CSCs. Then, we used autophagy analysis, RNA-Seq, and real-time PCR to identify the mechanism of the inhibition in LGR5(+) colorectal CSCs.

## Materials and methods

### Cell line and reagents

SW620 cell line (ATCC CCL-227) was obtained from the American Type Culture Collection (ATCC, Manassas, VA, USA). Recombinant human epidermal growth factor (EGF, Lot No.AF-100-15B) and recombinant human basic fibroblast growth factor (FGF, Lot No.AF-100-18B) were purchased from PeproTech, Inc. (Rocky Hill, NJ, USA). Dulbecco's modified Eagle's medium (DMEM)/F12 medium, B-27 (Lot No.17504-044), and N-2 (Lot No.17502-048) supplements and recombinant human leukemia inhibitory factor (LIF, Lot No. PHC9484) were purchased from Gibco (Life Technologies, Corp., Carlsbad, CA, USA). Curcumin (Lot No.C1386), hydroxychloroquine (HCQ, Lot No. PHR1782) and rapamycin (Lot No. V900930) were purchased from Sigma (Sigma-Aldrich, USA).

## Colorectal CSCs

SW620 cells (5 × 10^3^/well) were seeded in flat-bottom ultra-low attachment culture plates (Corning, NY, USA) in DMEM/F12 medium supplemented with 20 ng/mL EGF, 20 ng/mL FGF, B-27 (1 ×) supplement, N-2 (1 ×) supplement, and 10 ng/mL LIF. Then, the cells were incubated at 37 °C in a humidified incubator with 5% CO_2_ and 95% air [15]. After seven to ten days, sphere formations of colorectal CSCs were observed under DMi8 inverted light microscope (Leica Microsystems, Wetzlar, Germany).

### Fluorescence-activated cell sorting

Cells were labeled with anti-LGR5 MicroBeads (Lot No.5191031303, Miltenyi Biotec, Bergisch Gladbach, Germany) and then LGR5(+) cells were enriched by magnetic activated cell sorting. Fluorescence-activated cell sorting was performed using BD FACSAria Cell Sorter (BD Biosciences, San Jose, CA, USA) [16].

### Western blotting

The total protein of LGR5(+) SW620 CSCs, LGR5(-) SW620 CSCs, and SW620 CSCs was extracted, and 50 μg of the total protein was used for electrophoresis. We transferred the electrophoresis products onto polyvinylidene fluoride membranes, and the membranes were blocked with 5% skim milk for one hour. After washing with Tris-buffered saline with Tween-20 (TBST, 1 ×), the membrane was incubated with primary antibodies LGR5 (1:5000, Lot No. Ab75850; Abcam, Cambridge, UK) and β-tubulin (1:500, Lot No.K200059M; Solarbio, China), and shaken at 4 °C overnight. Next, the polyvinylidene fluoride membranes were washed thrice with TBST, incubated with a secondary antibody at room temperature for 1 h and washed with TBST again. Finally, immunoreactive products were visualized using enhanced chemiluminescence (Tanon-4200, China) and quantified using ImageJ software (version 1.50, National Institutes of Health).

### Sphere formation, cell viability, and cell apoptosis

LGR5(+) colorectal CSCs were incubated with different concentrations of curcumin (1 μM, 5 μM, 25 μM) for 48 h. Then, sphere formations of different groups were observed under inverted light microscope. The culture medium (DMEM/F12) was then discarded and 100 μL fresh culture medium was added, along with 10 μL CCK-8 reagent (Cell Counting Kit-8, Lot No.CK04; Dojindo Molecular Technologies, Inc., Tokyo, Japan) into each well, and incubated at 37 °C for one hour. The optical density was measured at 450 nm using a microplate reader (Multiskan Spectrum, Thermo, USA).

In this study, the Annexin V-APC (PI) Apoptosis Analysis Kit (Lot No.AO2001-11P-H, Tianjin Sungene Biotech Co., Ltd., Tianjin, China) was used to analyze cell apoptosis[17]. After treated with curcumin (5 μM) for 24 h, the LGR5(+) colorectal CSCs were suspended in 100 μL binding buffer, and stained with 5 μL APC-conjugated Annexin V and 5 μL propidium iodide for 15 min at room temperature in the dark. Finally, 400 μL binding buffer was added, and apoptotic cells were analyzed by flow cytometry (BD Biosciences, San Jose, CA, USA).

### Autophagy analysis

Because the green fluorescent protein (GFP) signals can be quenched and the monomeric red fluorescent protein (mRFP) signal is more stable in the acidic environment of the lysosome, autolysosomes, and autophagosomes were labeled with mRFP (red) or GFP (green), respectively[18]. The LGR5(+) colorectal CSCs were transfected with Ad-mCherry-GFP-LC3B (2.51 × 10^10^ PFU/mL), and treated with different concentrations of curcumin (1 μM and 5 μM) or rapamycin (100 nM) for 48 h. In this study, autophagy inducer rapamycin served as a positive control group. Then, the expression of mCherry and GFP was visualized using confocal fluorescence microscopy (Leica TCS SP8; Leica Microsystems) at 100 × magnification. Autophagy flux was evaluated by observing the number of yellow and red puncta.

After treated with curcumin (1 μM and 5 μM) or rapamycin (100 nM) for 24 h, the LGR5(+) colorectal CSCs were washed twice with ice-cold PBS, fixed using 2.5% glutaraldehyde for 60 min at room temperature, and post-fixed in 1% osmium tetroxide for 30 min. As described previously [19, 20], all samples were embedded in EPON resin and stained with uranyl acetate and lead citrate. The cells were observed under a transmission electron microscope (H-7650; Hitachi High-Technologies Corp., Tokyo, Japan).

According to our previous studies[18, 20], HCQ could inhibit tumor autophagy. To confirm the effect of curcumin-induced autophagy on curcumin-mediated cell inhibition of LGR5(+) colorectal CSCs, cells were co-treated and incubated with curcumin (5 μM) and HCQ (10 μM); sphere formation and cell viability were then analyzed.

### RNA-Seq for gene analysis

Total RNA of the LGR5(+) colorectal CSCs treated with and without curcumin (5 μM), were extracted using TRIzol® Reagent (Invitrogen, Carlsbad, CA, USA) according to the manufacturer's instructions. To explore the mechanism of curcumin in LGR5(+) colorectal CSCs, RNA-Seq was performed. Firstly, Agilent 2100 Bioanalyzer (Agilent Technologies, Santa Clara, CA, USA) was used to confirm RNA quality, then RNA-Seq libraries were prepared from three biological replicates and sequenced on an Illumina HiSeq (Illumina, Inc., San Diego, CA, USA). Fastqc and RSEM were used to assess the quality of the reads and calculate the expression of the genes, respectively [21]. Subsequently, the edgeR software (version 3.30.3) was used and the differential expression was calculated by Gene Read Count [22]. The screening criteria for significantly differentially expressed genes were FDR < 0.05 and |log_2_FC|≥ 1. These genes were analyzed by clustering expression patterns, and were classified according to the involved biological processes and molecular functions and associated cell components, using the Gene Ontology database. In addition, KOBAS (http://kobas.cbi.pku.edu.cn/home.do) was used to analyze the pathway enrichment analysis using Kyoto Encyclopedia of Genes and Genomes (KEGG).

Next, Cytoscape plugin iRegulon was used to identify master regulators that targeted these significantly expressed genes [23]. Transcription factors (TFs) of the master regulators were identified when they overlapped with the significantly expressed gene signatures. As previously described, the algorithm was based on a typical ranking-and-recovery strategy [24], and all the default parameters were left unchanged while predicting TFs. In this experiment, TFs with normalized enrichment score and normalized enrichment score (NES) ≥ 6 were set as the parameters to build the regulatory network.

### TFAP2A mRNA expression and prognosis correlation in CRC

A CRC cDNA microarray (MecDNA-HColA095Su01) containing 15 paired cancer and non-CRC tissues and 65 CRC tissues was purchased from Shanghai Outdo Biotech Company (Shanghai, China). The mRNA expression of *TFAP2A* and *GAPDH* was quantified with SYBR mixture (Takara Biotechnology, Co., Ltd., Dalian, China), using a real-time PCR machine (LightCycler 480 Instrument II, Roche Molecular Systems, Inc., Pleasanton, CA, USA). The primers used were the following: for *TFPA2A*, *TFAP2A* F: 5′-CGTGTCCCTGTCCAAGTCCAA-3′ and *TFAP2A* R: 5′-GACCCGGAACTGAACAGAAGA-3′ [25]; for human β-actin, Human β-actin-F1: 5′-GAAGAGCTACGAGCTGCCTGA-3′ and human β-actin-R1: 5′- CAGACAGCACTGTGTTGGCG-3'.

To investigate the correlation between *TFAP2A* expression with overall survival in CRC, the expression of *TFAP2A* was divided into two categories: high expression (_Δ_CT < 11.1) and low expression (_Δ_CT ≥ 11.1), and the correlation was analyzed by the Kaplan–Meier method with a log-rank test or Wilcoxon test.

### Statistical analysis

All data were expressed as the mean ± standard deviation of at least three individual experiments. The data were analyzed by one-way analysis of variance (ANOVA) using the SPSS Statistics 26.0 (IBM, Armonk, NY, USA). The following values were considered statistically significant: *p < 0.05, **p < 0.01, ***p < 0.001.

## Results

### LGR5(+) colorectal CSCs were obtained

**Fig. 1:**
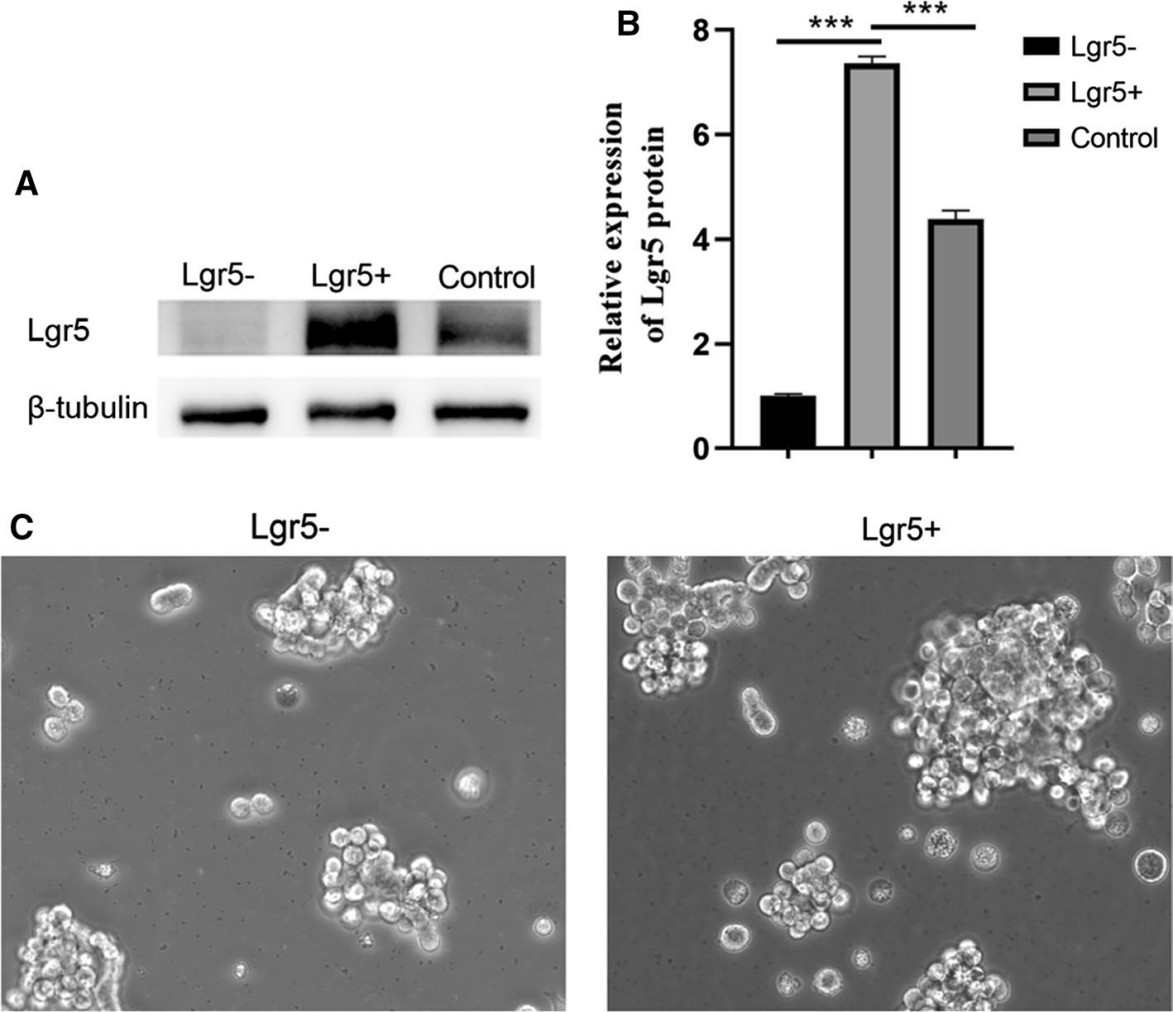
Identification of LGR5(+) colorectal CSCs. **a** The LGR5 protein expression of LGR5(+) SW620 CSCs, LGR5(−) SW620 CSCs, and SW620 CSCs was demonstrated by western blotting. **b** Relative expression levels of LGR5 in LGR5(+) SW620 CSCs, LGR5(−) SW620 CSCs, and SW620 CSCs. **c** Tumorsphere formation of LGR5(+) SW620 CSCs and LGR5(−) SW620 CSCs. ***p < 0.001

Human LGR5(+) colorectal CSC models provide an opportunity to study the effect and mechanism of antitumor drugs. SW620 cells were cultured in flat-bottom ultra-low attachment culture plates in DMEM/F12 medium supplemented with 20 ng/mL EGF, 20 ng/mL FGF, B-27 (1 ×) supplement, N-2 (1 ×) supplement, and 10 ng/mL LIF. After sphere formation was observed, cells were labeled with anti-LGR5 MicroBeads, then LGR5(+) cells were enriched by magnetic activated cell sorting. The results of western blotting revealed that LGR5 was highly expressed in LGR5(+) SW620 CSCs, but was hardly detected in LGR5(-) SW620 CSCs (Fig. 1a, b). In addition, the tumorsphere was better formed in LGR5(+) SW620 CSCs than in LGR5(-) SW620 CSCs (Fig. 1c).

### Curcumin inhibited tumorsphere formation, decreased cell viability and promoted apoptosis of LGR5(+) colorectal CSCs

**Fig. 2:**
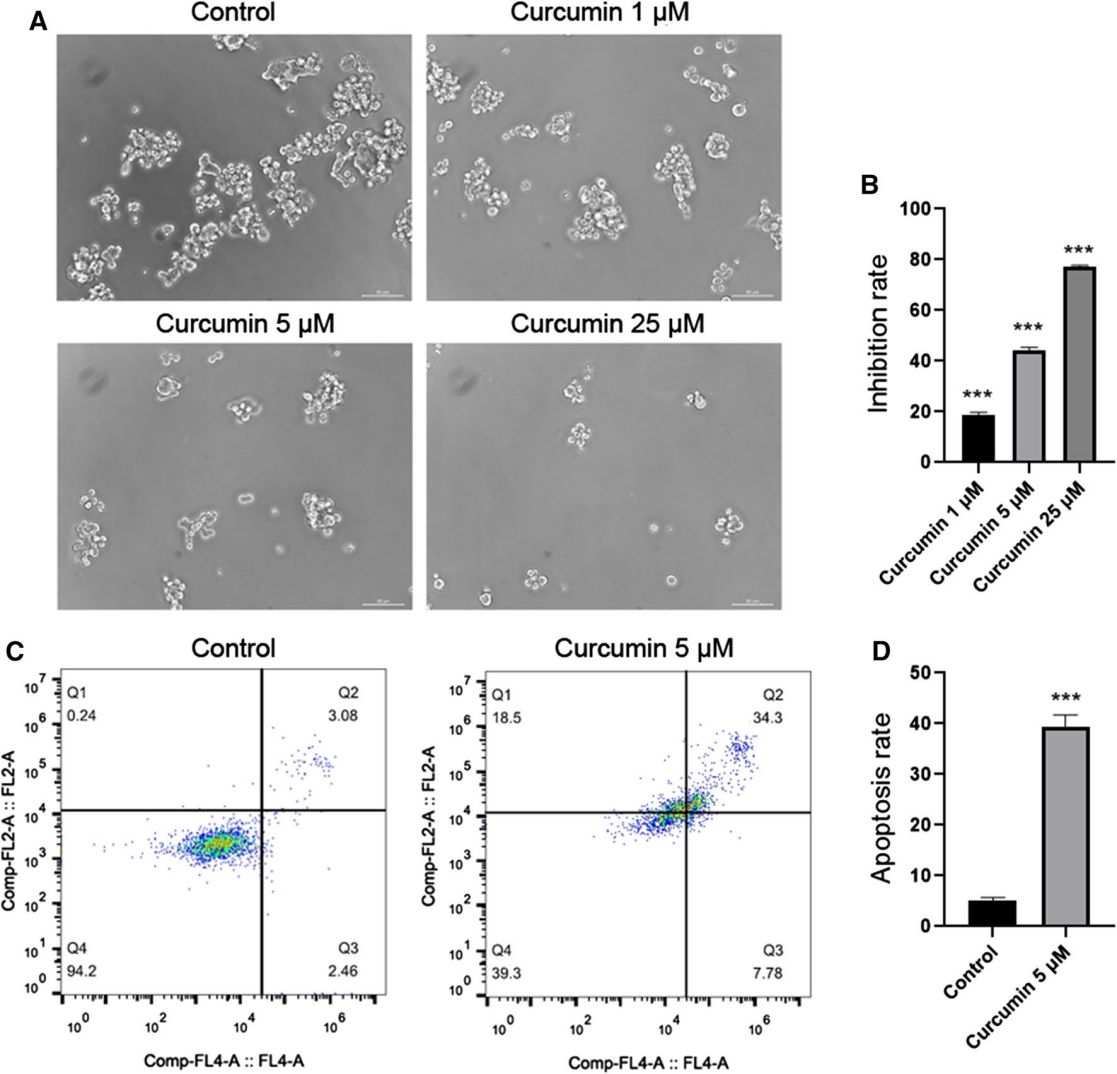
Curcumin suppressed LGR5(+) colorectal CSCs. **a** Curcumin reduced tumorsphere formation of LGR5(+) colorectal CSCs in a dose-dependent manner. **b** Cell viability showed that curcumin inhibited cell growth of LGR5(+) colorectal CSCs in a dose-dependent manner. **c**, **d** Cell apoptosis analysis of LGR5(+) colorectal CSCs treated with curcumin (5 μM). ***p < 0.001

To confirm the effect of curcumin in LGR5(+) colorectal CSCs, LGR5(+) SW620 CSCs were treated with curcumin (1 μM, 5 μM, and 25 μM). We found that curcumin reduced tumorsphere formation in a dose-dependent manner (Fig. 2a). The results of cell viability analysis showed that curcumin also inhibited the growth of LGR5(+) colorectal CSCs in a dose-dependent manner (Fig. 2b).

In apoptosis analysis, the apoptosis rate of cells treated with curcumin (5 μM) was 39.36 ± 2.36%, whereas the apoptosis rate of control cells was 5.07 ± 0.62% (Fig. 2c, d).

### Curcumin induced autophagy of LGR5(+) colorectal CSCs

**Fig. 3:**
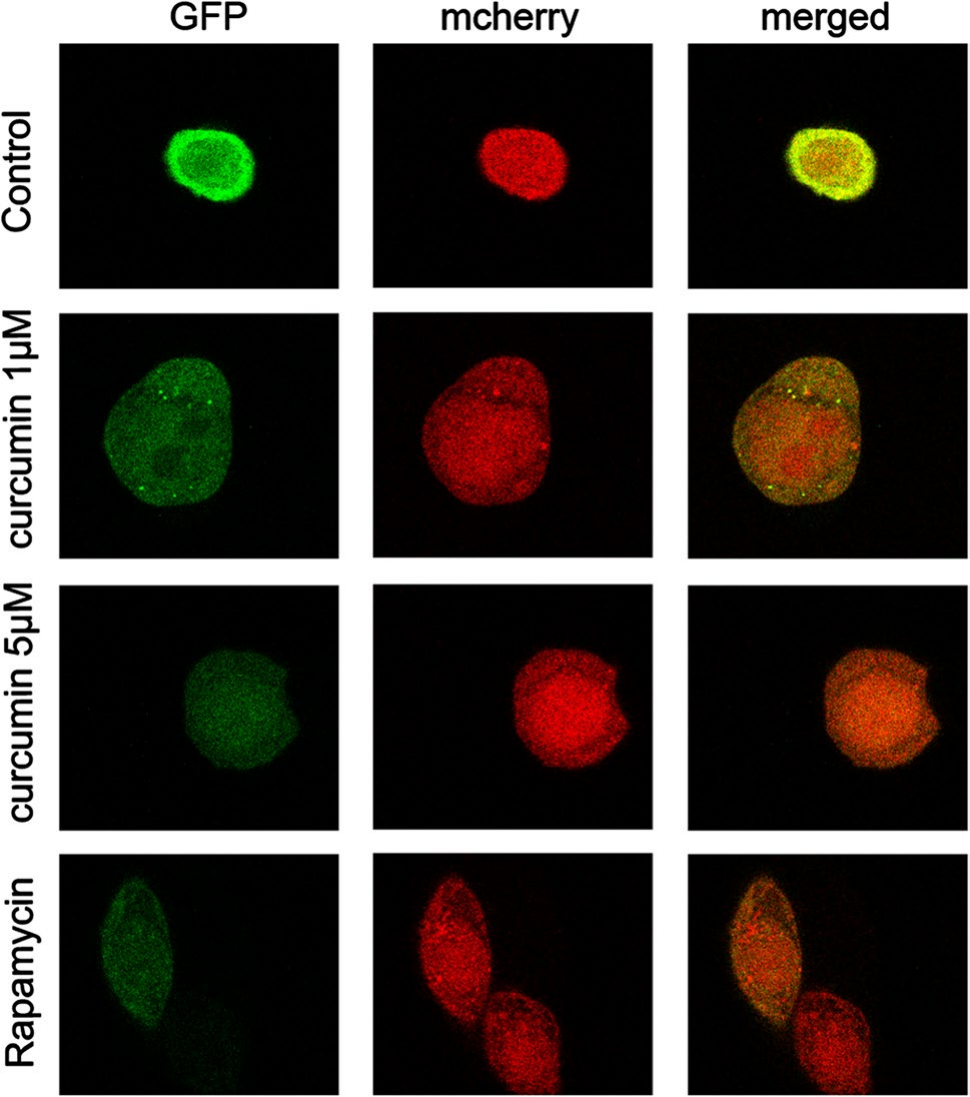
After the cells were treated with different concentrations of curcumin (1 μM and 5 μM), or rapamycin (100 nM), autophagy flux was evaluated by the mCherryEGFP-LC3 assay. Autolysosomes and autophagosomes were labeled with mRFP or GFP, respectively

**Fig. 4:**
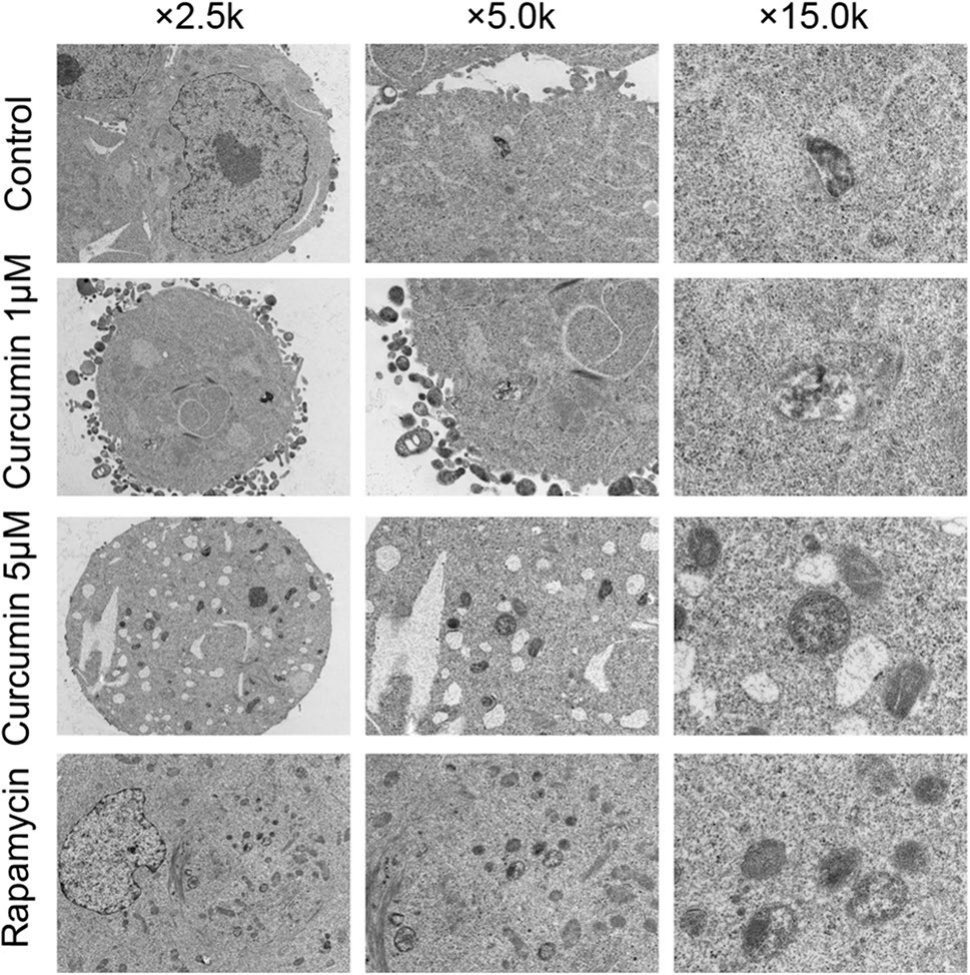
Cells were observed under a transmission electron microscope after the cells were treated with different concentrations of curcumin (1 μM and 5 μM), or rapamycin (100 nM)

Although it is known that curcumin can induce autophagy in many tumor cells, whether curcumin can induce autophagy of LGR5(+) colorectal CSCs was unclear. Thus, we transfected the curcumin (1 μM and 5 μM) treated-LGR5(+) colorectal CSCs with Ad-mRFP-GFP-LC3 adenovirus, and then detected the two different fluorescent signals to monitor the autophagic flux status post-treatment with curcumin. In this study, autophagy inducer rapamycin served as a positive control group, then we found both yellow and red puncta were increased in cells treated with either curcumin or rapamycin, indicating that curcumin can induce autophagy in LGR5(+) colorectal CSCs (Fig. 3). In addition, we found that the degree of autophagy was proportional to the concentration of curcumin. These results were consistent with the data from transmission electron micrographs (Fig. 4), suggesting that curcumin induced autophagy.

**Fig. 5:**
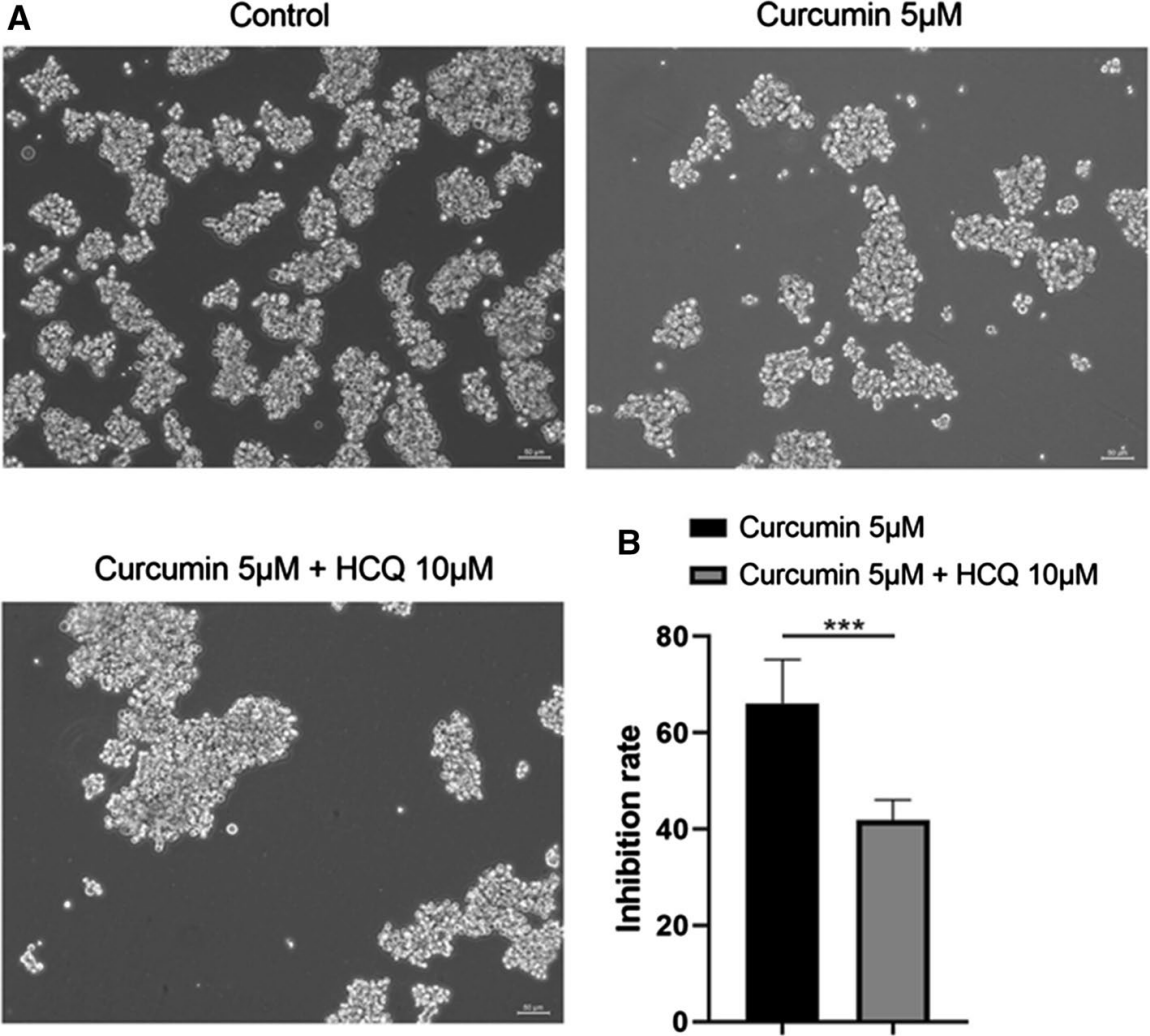
Effect of curcumin-induced cell proliferation inhibition was decreased on co-treatment of cells with the autophagy inhibitor, hydroxychloroquine (HCQ). **a** Sphere formation assay of LGR5(+) colorectal CSCs mono-treated with curcumin (5 μM) or co-treated with HCQ (10 μM). **b** In the CCK-8 assay, inhibition rate of LGR5(+) colorectal CSCs mono-treated with curcumin (5 μM) or co-treated with HCQ (10 μM). ***p < 0.001

As shown in Fig. 5a, co-treatment with curcumin (5 μM) and HCQ (10 μM) can increase the tumorsphere formation of LGR5(+) colorectal CSCs, compared to mono-treatment with curcumin. In the CCK-8 assay, inhibition rate decreased to 41.83% upon co-treatment with curcumin and HCQ, versus 66.12% upon mono-treatment with curcumin (Fig. 5b). Therefore, these results suggest that curcumin-mediated cell inhibition may be associated with curcumin-induced autophagy.

### Curcumin downregulated the oncogenic TFAP2A-mediated ECM pathway

**Fig. 6:**
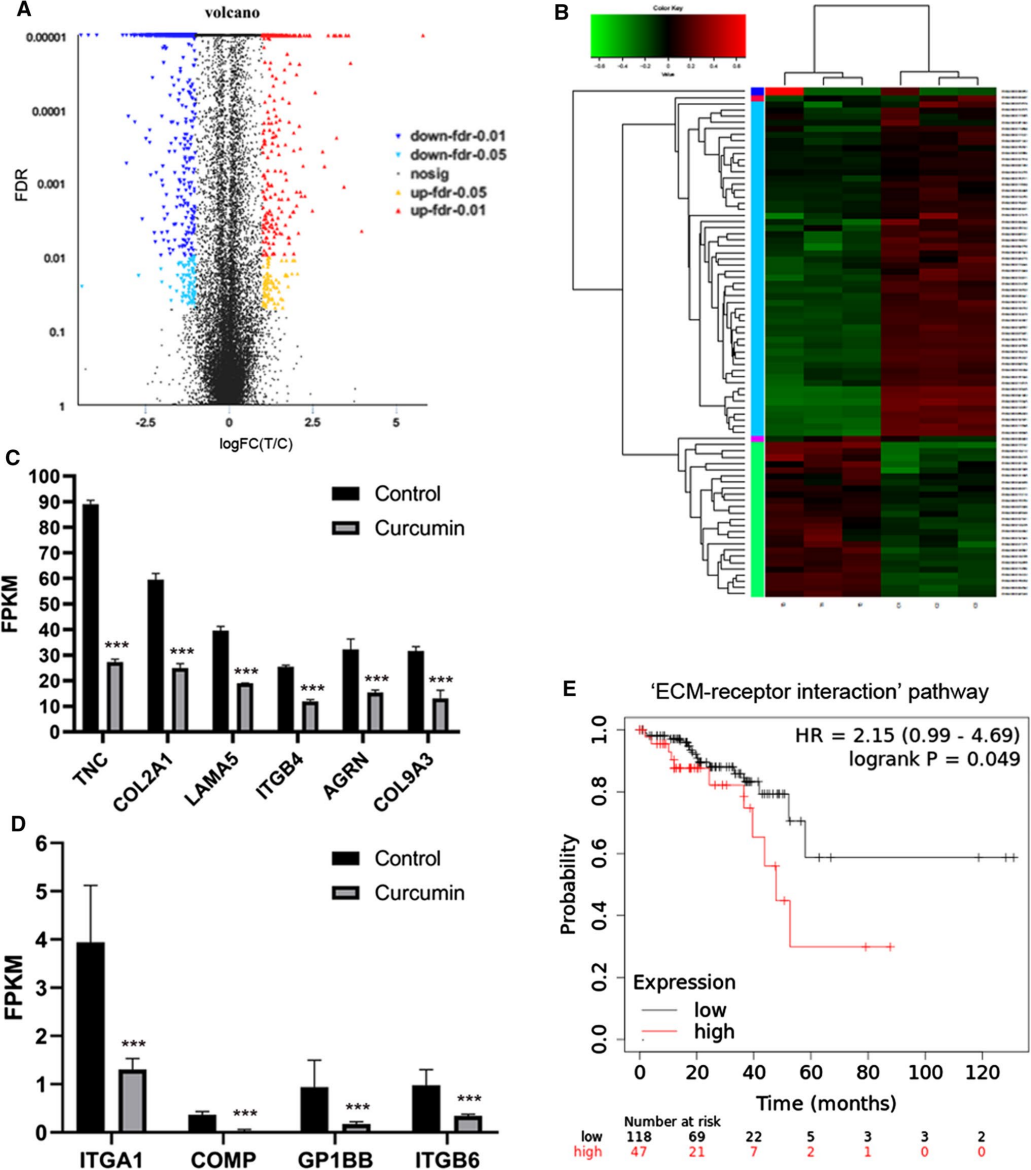
RNA-Seq analysis. **a** The volcano plot of the significantly differentially expressed genes between curcumin-treated cells and control cells. **b** Heat map of significantly differentially expressed genes in the top six pathways. **c**, **d** Ten significantly negatively expressed genes involved in ECM–receptor interaction, including *GP1BB*, *COL9A3, COMP, AGRN, ITGB4, LAMA5, COL2A1, ITGB6, ITGA1,* and *TNC*. **e** Kaplan–Meier survival curves of ECM–receptor interaction pathway including the ten significantly negatively expressed genes in CRC patients. *p < 0.05, **p < 0.01, ***p < 0.001

In order to identify mechanisms of curcumin in LGR5(+) colorectal CSCs, RNA-Seq was used, and the result revealed that there were 1098 significantly differentially expressed genes in curcumin-treated cells and control cells, with 363 upregulated and 735 downregulated genes. These significantly differentially expressed genes between curcumin-treated cells and control cells were shown in Fig. 6a, and heat map of significantly differentially expressed genes in the top six pathways was shown in Fig. 6b.

KEGG pathway analyses revealed differential expression in curcumin-treated LGR5(+) cells involved in ‘Pathways in cancer,’ ‘PI3K-Akt signaling pathway,’ ‘MAPK signaling pathway,’ ‘Wnt signaling pathway,’ ‘NF-kappa B signaling pathway,’ ‘ECM-receptor interaction,’ and ‘Lysosome’ (Data not shown). In addition, there were ten significantly negatively expressed genes involved in ECM-receptor interaction, including *GP1BB*, *COL9A3*, *COMP*, *AGRN*, *ITGB4*, *LAMA5*, *COL2A1*, *ITGB6*, *ITGA1*, and *TNC* (Fig. 6c, d). Using Kaplan–Meier Plotter (www.kmplot.com), we found that the ECM–receptor interaction pathway included these ten significantly negatively expressed genes and were associated with shorter survival of CRC patients (hazard ratios, HR = 2.15 (0.99 − 4.69); logrank *p* = 0.049) (Fig. 6E).

**Fig. 7:**
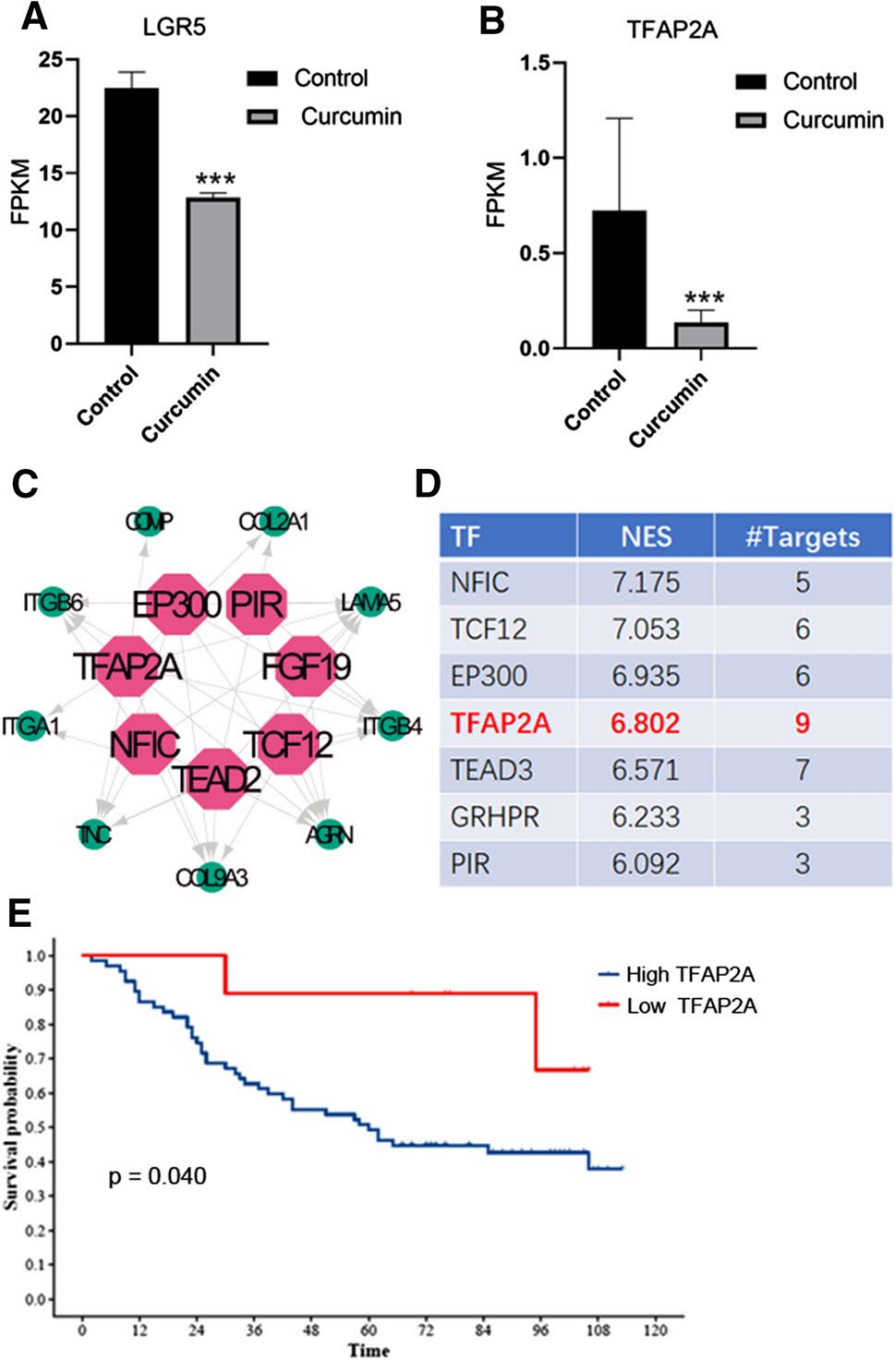
Curcumin suppressed LGR5(+) colorectal CSCs by transcriptionally repressing oncogenic *TFAP2A*. **a** Curcumin decreased the expression of *LGR5*. **b** Curcumin decreased the expression of *TFAP2A*. **c** Cytoscape plugin iRegulon was used to identify master regulators of targeted genes, and *TFAP2A* overlaps with nine genes in the regulatory network. **d** The normalized enrichment score (NES) of *TFAP2A* was 6.802. **e** *TFAP2A* expression was associated with poor survival for patients with CRC. *p < 0.05, **p < 0.01, ***p < 0.001

The results of RNA-Seq also revealed that the expression of LGR5 and *TFAP2A* in LGR5(+) colorectal CSCs treated with curcumin was significantly downregulated (Fig. 7a, b). Then, we used Cytoscape plugin iRegulon to identify the master regulators that targeted the aforementioned genes, and found the NES of *TFAP2A* was at 6.802, and as shown in the regulatory network, is a transcription factor. In addition, *TFAP2A* overlapped with nine out of ten genes, with the exception of *GP1BB* (Fig. 7c, d).

### TFAP2A was associated with poor survival in patients with CRC

To investigate the role of *TFAP2A* in CRC, the mRNA expression of *TFAP2A* in CRC and noncancerous colorectal tissues was quantified using real-time PCR. Our results showed higher *TFAP2A* mRNA expression in CRC tissues compared with that in noncancerous colorectal tissues, TFAP2A mRNA expression was not associated with age, gender, grade, and TNM stage (Table 1). After the expression of *TFAP2A* was divided into high and low expression categories, we further assessed the correlation between *TFAP2A* mRNA expression and survival of patients, and found that higher expression of *TFAP2A* was correlated with poor overall survival of CRC patients (Fig. 7e, *p* < 0.05).

**Table 1:** Correlation between *TFAP2A* expression and clinicopathological characteristics

	Variables	TFAP2A expression		Total	*χ*^2^	*p* value
		Low	High			
Age (year)					1.786	0.181
	≤ 65	7	32	39		
	> 65	2	35	37		
Gender					0.001	0.970
	Female	3	27	30		
	male	6	40	46		
Grade					0.032	0.857
	I/II	6	50	56		
	III	3	16	19		
T stage					0.012	0.912
	T2/T3	3	18	21		
	T4	5	45	50		
N stage					0.000	1.000
	N0	5	36	41		
	N1/N2	3	28	31		
M stage						1.000
	M0	9	66	75		
	M1	0	1	1		
TNM stage					0.000	1.000
	I/II	5	36	41		
	III/IV	3	28	31		

## Discussion

Colorectal cancer (CRC) is the world’s fourth most deadly cancer, and evidence shows that rare CSCs have the potential for self-renewal, proliferation, and differentiation in CRC [26]. LGR5 was originally isolated from colon cancer cells, where 56% LGR5(+) cells were observed in colorectal CSCs [27]. Importantly, few LGR5(+) colorectal CSCs could grow a tumor with the same phenotype as that of original cells, and silencing the LGR5 gene could increase the apoptosis of tumor cells [13]. Therefore, the importance of LGR5 as a stem cell marker gene of CRC is already well established [28], and we used LGR5(+) colorectal CSCs to investigate the effect and mechanism of curcumin.

In this study, we observed that curcumin inhibited tumorsphere formation, decreased cell viability in a dose-dependent manner. Curcumin also promoted apoptosis of LGR5(+) colorectal CSCs. In addition, curcumin can induce autophagy in many tumor cells. For example, curcumin inhibited proliferation, induced the autophagy and apoptosis in gastric cancer cells [29]. Similarly, our data suggest that curcumin increased tumor cell death partly by inducing autophagy, because the effect of curcumin-induced cell proliferation inhibition was decreased by co-treatment with the autophagy inhibitor, HCQ. Therefore, these results reveal that curcumin-induced autophagy may contribute to the antitumor effects of curcumin on LGR5(+) colorectal CSCs.

Next, the RNA-Seq data showed that curcumin decreased the expression of *GP1BB*, *COL9A3*, *COMP*, *AGRN*, *ITGB4*, *LAMA5*, *COL2A1*, *ITGB6*, *ITGA1*, and *TNC* in the ECM–receptor interaction pathway, which is also associated with shorter survival of CRC patients. As previously reported, the ECM acts as a structural support and provides stem cells with signals to regulate its function via reciprocal interactions between cells and the components of the ECM [30]. At present, many studies have confirmed that curcumin can regulate EMT pathway, for example, curcumin inhibited cancer cell invasion via downregulating MMPs and uPA [31]; curcumin exhibits antimetastatic properties of melanoma cells by regulating integrin receptors, collagenase activity, and expression of nonmetastatic gene 23 (Nm23), and E-cadherin [32]. However, the role of ECM in colorectal CSC remains unclear and will be investigated in future studies.

After identifying the master regulators that targeted the aforementioned genes, we found that curcumin inhibited LGR5(+) colorectal CSC proliferation by downregulating *TFAP2A*-mediated ECM-receptor interaction. *TFAP2A* is a member of the AP-2 family of transcription factors, and this family is composed of TFAP2A/AP-2α, TFAP2B/AP-2β, TFAP2C/AP-2γ, TFAP2D/AP-2δ, and TFAP2E/AP-2ε [33]. The aberrant expression of *TFAP2A* has been reported in different cancers [34]. For example, *TFAP2A* is overexpressed in human nasopharyngeal carcinoma and promotes tumorigenesis by influencing the HIF-1α/VEGF/PEDF pathway [35]. However, reduced *TFAP2A* expression is associated with poor prognosis in gastric cancer [36], and the loss of *TFAP2A* is linked with melanoma through the regulation of cell adhesion molecules [37]. In addition, Dimitrova found that the expression of *TFAP2A* is increased in more “stem-like” cancers [38]. Our results have shown that *TFAP2A* expression was markedly elevated in CRC compared with that in noncancerous colorectal tissues, and was associated with poor prognosis in CRC. Previous studies have found that curcumin induced apoptosis via reducing the expression of AP-2γ in human malignant testicular germ cells, whereas the pretreatment with the proteasome inhibitor MG132 blocked both the curcumin-induced reduction of AP-2γ and antiproliferative effect. Nevertheless, the effect of *TFAP2A* in CRC has not yet been reported. In future, the role of TFAP2A and other genes in curcumin-induced apoptosis of colorectal CSCs will be studied.

In summary, we found that curcumin suppresses the proliferation of LGR5(+) colorectal CSCs by inducing autophagy and transcriptionally repressing the oncogenic *TFAP2A*-mediated ECM pathway. Lastly, while our study entailed mostly in vitro experiments, further studies, especially in vivo, are needed to understand the mechanism of effect of curcumin in LGR5(+) colorectal CSCs and to evaluate this potential therapeutic approach to CRC. In addition, the correlation between *TFAP2A*-mediated ECM pathway and autophagy also deserves further study.
